# COVID-19 Vaccine Hesitancy among Unvaccinated Adults: A Cross-Sectional Exploratory Analysis of Vaccination Intentions in Italy Related to Fear of Infection

**DOI:** 10.3390/vaccines11121790

**Published:** 2023-11-30

**Authors:** Olivia Curzio, Liliana Cori, Fabrizio Bianchi, Federica Prinelli, Massimo Galli, Andrea Giacomelli, Maria Cristina Imiotti, Nithiya Jesuthasan, Virginia Recchia, Fulvio Adorni

**Affiliations:** 1 Institute of Clinical Physiology of the National Research Council (IFC-CNR), 56124 Pisa, Italy; olivia.curzio@ifc.cnr.it (O.C.); fabrizio.bianchi@ifc.cnr.it (F.B.); crisim@ifc.cnr.it (M.C.I.); virginia.recchia@cnr.it (V.R.); 2 Institute of Biomedical Technologies of the National Research Council (ITB-CNR), 20154 Segrate, Italy; federica.prinelli@itb.cnr.it (F.P.); nithiya.jesuthasan@itb.cnr.it (N.J.); 3 Third Division of Infectious Diseases, Fatebenefratelli Sacco Hospital, 20157 Milan, Italy; massimo.galli@unimi.it (M.G.); andrea.giacomelli@unimi.it (A.G.); fulvio.adorni@itb.cnr.it (F.A.)

**Keywords:** COVID-19, SARS-CoV-2, observational study, public health, self-reported data, web-based survey, vaccine hesitancy, risk communication, educational level, fear

## Abstract

From the initial COVID-19 outbreak, Italy was the first Western country to be seriously affected by the pandemic. Understanding vaccine hesitancy can help efforts to achieve broad vaccination coverage. The objectives of this research were to determine the extent of vaccine hesitancy in Italy and to understand the characteristics of those segments of the population with some hesitancy. Between January and February 2021, 41,473 subjects answered the second questionnaire delivered in phase II of the web-based EPICOVID19 survey. Among the included adult volunteers living in Italy, 4653 (11.2%) reported having previously received at least one dose of the COVID-19 vaccine. In the sample of 36,820 respondents, all not vaccinated (age 51.1 ± 13.5; 59.7% female; 63.6% high level of education), the comparison between hesitant and inclined participants was accompanied by percentages and odds ratios. A total of 2449 individuals were hesitant (6.7% of the unvaccinated ones). Hesitancy was higher among women (OR = 1.48; 95%CI: 1.36–1.62); it was highest in the 50–59 and 40–49 age groups and among those with a lower educational level. A higher level of education was associated with a lower proportion of hesitancy (5.54%) compared with 9.44% among respondents with a low level of education (OR = 0.56; 95%CI: 0.46–0.68). Hesitancy was most common in subjects who did not report fear of infection (12.4%, OR = 4.0; 95%CI: 3.46–4.61). The results can guide the design of tailored information and communication campaigns through considering objective and subjective characteristics.

## 1. Introduction

A total of 386,548,962 cases of infection and 5,705,754 deaths reported up to February 2022 were caused by the COVID-19 pandemic [1]. From the disease outbreak in February 2020 to 20 December 2021, with 5,389,155 confirmed cases and 135,641 deaths, Italy was the first Western country to be severely affected by the COVID-19 pandemic [2]. Only 57 countries have vaccinated 70% of their population against COVID-19. Despite the initiation of vaccination campaigns, attention to vaccine hesitancy (VH) by individuals is also growing.

To identify the extent of this problem, our study involves the identification of various factors, in a preliminary analysis, which could hinder the vaccination campaign. In a systematic review carried out in PubMed on 25 December 2020, survey studies on COVID-19 vaccine acceptance rates were analyzed from 33 different countries. The highest rates of COVID-19 vaccine acceptance among adults from the general population were found in Ecuador (97.0%), Malaysia (94.3%), Indonesia (93.3%), China (91.3%), and Italy (53.7%) [3].

Further and more in-depth studies are recommended internationally to address the problem of COVID-19 VH. Indeed, addressing the extent of COVID-19 VH in various countries is listed as a top priority to build confidence in COVID-19 vaccination efforts [3,4].

Previous research points to gender differences in COVID-19 VH. The health crisis has become gender-sensitive, including, but not limited to VH [5]. Based on our knowledge and in relation to previous scientific literature, our research hypothesis is that there are important differences in the level of hesitancy about vaccination by gender, age, educational level, and fear of contagion of each person.

This research is focused on the need for public health communication and it examines whether or not unvaccinated people with specific sociodemographic characteristics are willing to get vaccinated against COVID-19. Based on the responses of those who participated in phase II of the Italian EPICOVID19 web-based survey between January and February 2021, the objectives of this research were as follows: to understand the characteristics of those segments of the population with some degree of reluctance toward vaccination and determine the extent of vaccine hesitancy in Italy; to understand the characteristics of the most hesitant subjects; and to investigate the association between the demographic, social, and attitudinal characteristics of the population groups who are reluctant to get vaccinated against SARS-CoV-2.

The findings should guide the development of appropriate strategies for increasing vaccine uptake [6] and educational activities in preparation for vaccination campaigns, taking account of individual objective and subjective characteristics, in particular the fear of contagion. The results of this preliminary study will help legislators develop informed policies and make responsible health decisions for a beneficial vaccination campaign.

## 2. Materials and Methods

EPICOVID19 is a web-based survey with a cross-sectional phase I research design [7] and a longitudinal phase II research design, conducted on a sample of adult volunteers (18+ years) residing in Italy.

The participants were asked to complete the two questionnaires (phase I and II) after providing consent for participation. An earlier paper provided a description of the first questionnaire’s content [7] and the phase II questionnaire—used in the present research—was described in a previous paper [8]. Both questionnaires are easily available in the supplementary materials attached to the two mentioned research articles.

Online participatory surveys offered an important opportunity to collect individual-level data from large samples during the isolation period. In the two-phase EPICOVID19 survey, adult volunteers living in Italy during the first (April–May 2020) and second waves (January–February 2021) of the pandemic were enrolled. Validated scales and ad hoc questionnaires were used to collect socio-demographic, medical, and behavioural characteristics. Among those who provided e-mail addresses during phase I (105,355), 41,473 participated in phase II (mean age 50.7 years ± 13.5 SD, 60.6% female).

The study design and data were registered on ClinicalTrials.gov (https://clinicaltrials.gov/ct2/show/NCT04471701, accessed 18 December 2021). The EPICOVID19 study started as a collaborative project of a working group including epidemiologists, infectious disease physicians, biostatisticians, and public health professionals to improve knowledge on SARS-CoV-2 infection. The survey was designed after an extensive review of the existing literature. Most of the questionnaire items were chosen on the basis of standardized scales. The two surveys were conducted using the open-source management tool EUSurvey (https://ec.europa.eu/eusurvey, accessed 18 December 2021). The link to the first questionnaire was shared from 13 April to 2 June 2020 (https://epicovid19.itb.cnr.it/, accessed 30 December 2022).

The inclusion criteria were age ≥ 18 years, access to a mobile phone/computer/tablet with Internet connectivity, and consent to participate. In total, 207,341 participants clicked on the first link in the questionnaire and 198,822 provided consent to participate. Participants who consented to be contacted (n = 105,355, 53%) by providing their personal e-mail address during the first survey received an e-mail invitation (from 15 January to 28 February 2021) containing a link allowing them to complete the second questionnaire. Those who did not complete the EPICOVID19 phase II questionnaire within fifteen days of the invitation were sent up to three reminder e-mails. In total, 41,473 participants were included in this study.

The questionnaire collects individual information about COVID-19 infection, also including: socio-demographic features (age, education, employment, job position, at-risk due to infection, and socio-economic status); body mass index (BMI); number of chronic diseases; smoking habits; alcohol consumption; self-perceived health status; individual level deprivation [9]; personal stress [10]; and the feeling of being informed about COVID-19. Fear of contagion for oneself or relatives and fear for personal and relatives’ economic situation were assessed using a short questionnaire developed ad hoc for this survey [11].

The questionnaire consists of a total of 75 questions, each divided into several subsections. Variables of interest for the present study were the following: age, sex, pregnancy status, education, fear of contagion for self or relatives, and anti-COVID-19 vaccination(s) [11]. The already vaccinated subjects were excluded from the analyses. The propensity to vaccination regards the likelihood of being vaccinated in the future and was investigated through a specific question: “Thinking about the COVID-19 vaccine: I will definitely get the vaccine/I will probably get the vaccine, but before that I will inform myself better/I will probably not get the vaccine, but in any case, I will inform myself better/I will not get the vaccine/I can’t answer at this moment”. Two levels of hesitancy/propensity to vaccination were considered when analyzing the data: “I think I will not vaccinate”—and “Yes, I will vaccinate” or “I will probably vaccinate”. Hesitancy was determined by referring to this question only, which is considered sufficient in the overall balance of this exploratory analysis.

A descriptive analysis was conducted and each association between hesitation and the above-mentioned variables was expressed as an odds ratio (OR), with 95% confidence intervals (CIs). The analyses, including the multivariate logistic regression, were performed using Stata 15.0 version, and a two-sided *p*-value < 0.05 was considered statistically significant (StataCorp LP, College Station, TX, USA).

## 3. Results

There were 2449 hesitant individuals (6.7%) out of 36,820 unvaccinated respondents. The number of women who responded was higher than the number of men (21,987 vs. 14,853), and females were more likely to be hesitant about the vaccine (7.6%) than males (5.3%, OR = 1.48; 95%CI: 1.36–1.62). Thus, males were more likely to want to get vaccinated than their female counterparts in the sample examined. Among the women interviewed, those pregnant in particular were the most hesitant about vaccination (12.61% of pregnant women; OR = 2.60; 95%CI: 2.00–3.38) (Table 1).

Considering five age classes, hesitancy was higher in the classes “50–59” and “40–49” (8.6% and 8.4%, OR = 2.32; 95%CI: 1.86–2.90 and OR = 2.26; 95%CI: 1.80–2.83, respectively), intermediate in the class “30–39” (6.5%), and lower in the class “19–29” (3.9%). Vaccine hesitancy was quite low in percentage even among older people, the age class “≥60” (4.3%), but the association did not emerge as statistically significant (OR = 1.1, 95%CI: 0.87–1.38) (Table 1).

Hesitancy was higher among subjects with a lower educational level (9.4%), lower when the educational level was intermediate (8.49%; OR = 0.89, 95%CI: 0.73–1.08), and much lower among the respondents with a high educational level (5.5%; OR = 0.54, 95%CI: 0.46–0.68) (Table 1).

Investigating the fear of being infected with SARS-CoV-2 for oneself, neutral subjects and individuals who declared little or no fear had a higher proportion of hesitant individuals than those who had a lot of fear. Compared to the percentage of low hesitancy among those afraid of being infected (3.43%), the percentage increases as the fear decreases: 7.02% among neutral and 12.43% among a little or not at all frightened (OR = 2.13; 95%CI: 1.81–2.50 and OR = 4.0; 96%CI: 3.46–4.61, respectively) (Table 1).

When investigating fear for a family member, the reverse trend between fear and hesitancy described above emerges even more strongly, with a similar pattern to fear for oneself.

The multivariate logistic analysis—including in the model as potential confounders all of the variables considered in this study—substantially confirmed the results of the univariate analyses, both with regard to the confounders and to the two fear of being infected variables. For the latter, in comparison with the results of the univariate analyses, the multivariate analysis shows slightly lower (though still high and significant) probability of being uncertain about vaccination among those declaring little or no fear of being infected for themselves (OR 3.6; 2.9–4.5) and for their family (OR 3.0; 2.4–3.8), and also among those who declared themselves to be neutral ((OR 1.9; 1.6–2.4) and (1.9; 1.6–2.3), respectively), in comparison with the very frightened ones considered as a reference (OR = 1.0).

## 4. Discussion

Vaccination has emerged as the most cost-effective public health strategy to maintain population health, with various social and economic benefits. From the beginning of the pandemic, it was clear that vaccination would be the best tool for limiting the spread of the disease globally. In Italy, vaccinations began to be available and easily accessible at the very beginning of the year 2021, the time period in which the information contained in this brief report was gathered through a questionnaire. On 1 January 2021, there were 11,251 vaccine administrations; on 1 February 2021, there were 4344 first doses and 81,895 second doses, with the cumulative administration of 731,579 vaccine doses; on 1 March 2021, there were 109,718 first doses and 24,526 second doses, with the cumulative administration of 136,178. One year after—on 1 March 2022—the cumulative vaccine administration was 87,020,880. Due to the rapid growth of vaccination, also generated by an intensive information campaign, the data collected in January and February 2021 by the EPICOVID19 questionnaire are of crucial importance to better understand the factors that functioned as barriers or facilitators towards vaccination (https://github.com/italia/covid19-opendata-vaccini, accessed on 1 January 2023).

The widespread acceptance of vaccines is necessary to achieve adequate immunisation coverage. We found that a small percentage of the sample of the unvaccinated respondents were hesitant to vaccinate (6.7%); this sub-population is of interest in understanding how best to set up information, education, and awareness campaigns that further reduce this percentage [12]. In the present study, it was shown that the considered factors influence vaccination readiness (gender, age, education, pregnancy, and fear of contagion), as previously found in research in other countries. Vaccine hesitancy, a state of indecision regarding a vaccination decision, is conceptualized in the literature as involving (1) cognition or affect, (2) behaviour, and (3) decision-making. Data obtained from this research show that women’s hesitancy is greater than men’s, as has been similarly established in other studies carried out in some European countries, such as Spain, Germany, Sweden, and the UK [13]. According to our findings, women who are hesitant are more likely to be between 40 and 59 years old. Previous European studies do not support this result, since they showed that younger women were more sceptical about the COVID-19 vaccine [14]. These studies also suggested that fake news widely shared on social media played a negative role in highlighting the possible dangers of COVID-19 vaccines on the menstrual cycle, fertility, pregnancy, and breastfeeding [15]. Additionally, our data indicate that the percentage of women who are hesitant to receive the COVID-19 vaccine increases in pregnancy from 7.46% to 12.61%. Accordingly, data collected from Belgium, Norway, the Netherlands, Switzerland, Ireland, the UK [16], and Italy [17] showed that hesitancy towards the COVID-19 vaccine might occur more often in pregnancy. This is not unexpected, as previous studies have shown that women are less likely to use drugs during pregnancy [18,19]. Moreover, the state of pregnancy itself—which is mostly considered as a factor to refuse any vaccine [20,21]—is combined with the lack of certainty and robust scientific evidence specifically regarding the COVID-19 vaccine.

Consistent with prior scientific evidence—obtained in France, Germany, Spain, Sweden, the Netherlands, and Italy itself—a higher proportion of hesitant people emerge among groups with a low level of education. All of these results showed that vaccine hesitancy is estimated to be lower for individuals with post-secondary education compared to the reference group reaching a level of education up to secondary [22,23]. Steinert and colleagues (2022) [24] investigated the heterogeneity of hesitancy toward the COVID-19 vaccine across eight European countries. Despite the percentage of adults who reported being hesitant ranging from 6.4 percent in Spain to 61.8 percent in Bulgaria, some determinants were found to be consistent among different countries. In five of them, women appear to be more hesitant to receive the COVID-19 vaccine than men. In some countries, hesitancy towards the vaccine decreased with increasing education. Age and education were inversely related to vaccine hesitancy in the present analysis, but they were probably not linear and dissimilar across European countries. When combined with our own research, all of these results showed that the current body of literature highlights the absence of a definitive consensus on the correlation between age, educational level, and vaccine hesitancy. Deep understanding of the factors contributing to a favourable or negative attitude toward COVID 19 vaccination should be sought in future research [24]. Toshkov (2023) [25] used comparative data from 27 European countries to examine predictors of COVID-19 vaccine hesitancy. Despite the heterogeneity of hesitancy in the included countries, the analyses showed that several demographic factors have significant associations with vaccine hesitancy and refusal. Age and education were inversely related to vaccine hesitancy in the bivariate analysis, but they were probably not linear and dissimilar across European countries. Males, urban dwellers, and office workers were less likely to be vaccine hesitant. The author concludes his extensive and thorough analysis by emphasizing that many variables and the specificity of each area must be considered. The patterns that explain vaccine hesitancy do not seem to differ much across regions. According to Toshkov’s analysis, other individual variables that have a bearing on vaccine hesitancy are related to distrust of national governments and physicians as sources of relevant information [25].

When analyzing fear [11], the results become particularly interesting and suggestive, especially for understanding the factors driving vaccination acceptance or refusal [26]. Fear of COVID-19 may play a significant role in reducing vaccine hesitancy [27]. In the results of the present study, fear of contagion is one of the psychological states most correlated with the propensity to vaccinate, or a real situation in which people find themselves in a work or social context. Hesitancy towards vaccines decreases as fear increases. Fear of infecting family members [11] leads to a greater willingness to get vaccinated and thus less hesitation.

A wide variety of methods have been used to measure vaccine hesitancy [28]. Reasons for hesitation include the safety and efficacy of vaccination, considering vaccination unnecessary, and trust issues regarding biomedical research [29] and the health and surveillance system [3,30,31]. One of the most common reasons for vaccine hesitancy is a lack of confidence in the COVID-19 vaccine. Vaccine hesitancy is predicted by lower fear of experiencing COVID-19 [32], unstable employment status, decreased family income, and worsening health status [33,34]. The reasons for vaccine hesitancy appear to be linked to a complex interrelated network of factors [35]. Even with compulsory vaccination, the hesitancy toward COVID-19 vaccines remains a concern among healthcare workers, both because of the type of work they carry out daily and because of their role in promoting vaccination among patients and communities.

### Limitations

In this study, only one variable regards the propensity to vaccinate, since it is enclosed within a questionnaire conceived for a broad-based survey. Nevertheless, we are confident that the investigated associations can enhance knowledge about the vaccine hesitancy related to specific target groups and to their risk perception, providing hints for further investigations through specific surveys. As explained above, this brief report addresses the research hypothesis that there are important differences in hesitancy about vaccination among individuals, depending on their gender, age, educational level and fear of contagion. In future studies, further characteristics should be investigated to better inform health professionals, risk managers, and decision-makers. They are, for instance, unstable employment status, decreased family income, and worsening health status as predictive factors for vaccine hesitancy [33,34], as well as their mutual relations. In this context, the same research group is currently working on a multivariate analysis that considers more variables than those that have been investigated here. To have a more solid basis in developing guidelines to address vaccine hesitancy, future research should also probe the causal nature of the associations explored in these preliminary findings. Actually, the observational nature of the evidence presented here precludes causal claims. Moreover, future research should attempt to link the individual characteristics to more general social and political attitudes [25].

## 5. Conclusions

The increasing challenges regarding vaccination coverage need to be addressed by public health policies. This is crucial in the current phase in which there is a slow resurgence of COVID-19 cases, while new variants of the virus are emerging worldwide. Accordingly, work is being carried out on the identification and distribution of further vaccines, with all the difficulties associated with their social acceptance. The factors related to sociodemographic characteristics leading to vaccine acceptance, hesitation, or rejection are complex. In order to support policy, it is essential that scientific evidence be produced on these aspects. The main message of the analysis is that people who do not think they or their families are at risk of contracting COVID-19 are more likely to be hesitant about the vaccine. It is important to underline the importance of educating people about the disease that the vaccine aims to prevent as well as the importance of educational activities based on communication about the vaccine itself.

Communication strategies need to be targeted and accompanied by insights into risk perception to test their impact [36]. To promote confidence in vaccines and create tailor-made strategies, it is important to understand the sociodemographic characteristics of people who feel hesitant about the vaccine, their fear of contagion, and why they feel hesitant.

Given the results presented here, more attention should likely be paid to information campaigns for the female population—particularly during pregnancy—and to people with a low level of education. Communication/educational initiatives should be designed considering specific problems faced in particular by groups of the population with a low level of education and who are affected by an unequal distribution of power and resources. In Italy, a specific plan must be developed to reinforce public health literacy and trust in health institutions, as underlined in several research studies developed in the country [37].

Campaigns must be informative regarding specific issues, facilitating choices and contributing to increased awareness. It is often not enough to provide more information on the risks and benefits of the vaccine [38]. The goal of public health intervention for outright vaccine rejecters should focus on minimizing the effect their critical speeches might have on others [39]. On the other hand, it would be important to maintain trust in vaccines in people who accept vaccination through a simple and transparent type of communication. The WHO defined the essential determinants of trust by summarizing six capacities of people when involved in communication: competence, objectivity, fairness, consistency, sincerity, and faith, which must be translated to public education [40,41,42]. Promoting the sharing of personal stories about the COVID-19 vaccine on social media, combined with interventions targeting specific reasons for hesitancy towards the COVID-19 vaccine and emphasising freedom from fear once vaccinated, could be effective in reducing hesitancy [43].

Ultimately, to enhance trust in institutions, the public must be adequately informed, and this means reaching specific target groups that are more likely to be hesitant towards vaccination. This main objective can only be pursued through communication campaigns with monitored achievements and through communicators who are supported with continuous data about hesitant target groups. This approach could ensure the increased effectiveness of public health policies over time, especially with regard to COVID-19 or any future pandemics of significant importance.

## Figures and Tables

**Table 1:** Propensity to SARS-CoV-2 vaccination and vaccine hesitancy, sociodemographic features, and fear.

	Propensity to Vaccination		Hesitancy			
	Yes (n.) *	Not (n.) **	(%)	Odds Ratio	95% CI	
Total	34,371	2449	6.65			
Sex						
Male	14,072	781	5.26	1.00	1	
Female	20,299	1668	7.59	1.48	1.36	1.62
Not pregnant	19,814	1598	7.46	1.45	1.33	1.59
Pregnant	485	70	12.61	2.60	2.00	3.38
Age						
19–29	2208	90	3.92	1.00	1	
30–39	5699	397	6.51	1.71	1.35	2.16
40–49	7308	672	8.42	2.26	1.80	2.83
50–59	8699	822	8.63	2.32	1.86	2.90
60+	10,457	468	4.28	1.10	0.87	1.38
Education ***						
Low	1180	123	9.44	1.00	1	
Intermediate	11,085	1029	8.49	0.89	0.73	1.08
High	22,106	1297	5.54	0.56	0.46	0.68
Fear of being infected						
Yes, a lot	6502	231	3.43	1.00	1	
Quite enough	12,521	466	3.59	1.05	0.89	1.23
Neutral	6422	485	7.02	2.13	1.81	2.50
Little or no fear	8926	1267	12.43	4.00	3.46	4.61
Fear of infection for FMs						
Yes, a lot	14,697	649	4.23	1.00	1	
Quite enough	12,591	717	5.39	1.29	1.16	1.44
Neutral	3154	338	9.68	2.43	2.12	2.78
Little or no fear	3929	745	15.94	4.29	3.84	4.80

Notes: Propensity to vaccinate: * yes or probably yes/** not or probably not. FMs: family members; *** education: low = none or primary school; intermediate: middle high school or high school; high: university degree or postgraduate degree (Ph.D., vocational master’s degree, or medical specialization).

## Data Availability

The data presented in this study are available on request from the corresponding author.

## Trial Registration

ClinicalTrials.gov NCT04471701; https://clinicaltrials.gov/ct2/show/NCT04471701, accessed on 12 February 2021.
